# GoPeaks: histone modification peak calling for CUT&Tag

**DOI:** 10.1186/s13059-022-02707-w

**Published:** 2022-07-04

**Authors:** William M. Yashar, Garth Kong, Jake VanCampen, Brittany M. Curtiss, Daniel J. Coleman, Lucia Carbone, Galip Gürkan Yardimci, Julia E. Maxson, Theodore P. Braun

**Affiliations:** 1 Knight Cancer Institute, Oregon Health & Science University, Portland, USA; 2 Department of Biomedical Engineering, Oregon Health & Science University, Portland, USA; 3 Knight Cardiovascular Institute, Oregon Health & Science University, Portland, USA; 4 Center for Early Cancer Detection, Oregon Health & Science University, Portland, USA; 5 Division of Oncologic Sciences, Oregon Health & Science University, Portland, USA; 6 Division of Hematology & Medical Oncology, Oregon Health & Science University, Portland, USA

**Keywords:** GoPeaks, CUT&Tag, Histone modifications, Peak calling, Epigenetics, CUT&RUN, ChIP-seq, ATAC-seq, MACS2, SEACR

## Abstract

Genome-wide mapping of histone modifications is critical to understanding transcriptional regulation. CUT&Tag is a new method for profiling histone modifications, offering improved sensitivity and decreased cost compared with ChIP-seq. Here, we present GoPeaks, a peak calling method specifically designed for histone modification CUT&Tag data. We compare the performance of GoPeaks against commonly used peak calling algorithms to detect histone modifications that display a range of peak profiles and are frequently used in epigenetic studies. We find that GoPeaks robustly detects genome-wide histone modifications and, notably, identifies a substantial number of H3K27ac peaks with improved sensitivity compared to other standard algorithms.

**Supplementary Information:**

The online version contains supplementary material available at 10.1186/s13059-022-02707-w.

## Background

Modification of histone proteins is a key mechanism of transcriptional regulation [1]. Histones package eukaryotic DNA into chromatin and control DNA conformation and organization [2]. Modification of the histone protein results in alteration of chromatin structure and recruitment of nuclear proteins to genomic features [3, 4]. For example, trimethylation of histone 3 lysine 4 (H3K4me3) facilitates the binding of positive transcriptional regulators to transcription start sites [5–7]. Similarly, acetylation of histone 3 lysine 27 (H3K27ac) neutralizes the positive charge of the histone tail and loosens the interaction between nucleosomes and DNA, allowing access of transcription factors to DNA regulatory sequences [8, 9]. Transcription factors are canonical regulators of transcription whose binding is strongly correlated with histone modifications [4, 10–12]. Large-scale studies have demonstrated that transcription factor-binding profiles can be used to predict histone modifications [13]. An understanding of genome-wide histone modifications is crucial to the understanding of transcriptional regulation.

Chromatin immunoprecipitation with sequencing (ChIP-seq), which couples antibodies that recognize DNA-associated proteins with next-generation sequencing technology, has enabled genome-wide profiling of histone modifications and transcription factors [5, 14–16]. Although widely used for epigenetic profiling, ChIP-seq is prone to high background, artificial enrichment of highly expressed genes, and often requires a prohibitively large number of cells per experiment [17, 18]. Enzyme-tethering strategies including Cleavage Under Targets and Tagmentation (CUT&Tag) and Cleavage Under Targets and Release Using Nuclease (CUT&RUN) have been developed to overcome these issues and perform epigenetic profiling with a low number of cells and minimal background [19–22].

**Fig. 1:**
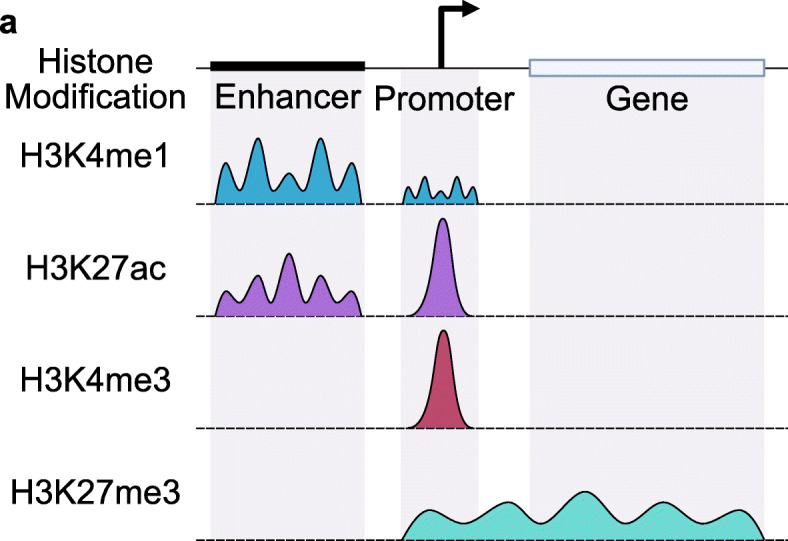
Histone modifications exhibit a range of peak profiles. Representative peak profiles for H3K4me1, H3K27ac, H3K4me3, and H3K27me3 histone modifications. Black box indicates an enhancer region and the gray box indicates the gene body

Epigenetic studies require mapping multiple histone modifications for a comprehensive understanding of transcriptional regulation. Detecting regions bound to lysine 4 residues on histone 3 that are mono- (H3K4me1) or trimethylated aids with the identification of promoters and enhancers, respectively, throughout the genome [5, 23–26]. Co-localization of H3K4me1 and H3K4me3 with H3K27ac is characteristic of activated genomic features [27, 28]. Histone modifications can also be associated with heterochromatic regions of the genome where gene transcription is repressed. Localization of trimethylation of histone 3 lysine 27 (H3K27me3), for example, is associated with promoters and gene bodies of silenced genes [5, 29–32]. Regions of modified histones in ChIP-seq and CUT&Tag are identified as stacks of aligned reads; such regions are called peaks. The peak profiles of common histone modifications are highly variable, so algorithms that identify histone modification peaks need to robustly detect a range of peak profiles. While H3K4me3 peaks tend to be sharply localized, H3K4me1 and H3K27me3 peaks span a broader domain (Fig. 1) [30–34]. Moreover, H3K27ac can mark large domains such as super-enhancers as well as smaller, discrete regions such as promoters, thus having both broad and narrow characteristics [25, 32–36]. In order to extract meaning for epigenetic studies reliant on histone modification CUT&Tag datasets, peak calling algorithms need to be flexible to identifying narrow and broad peak characteristics.

Peak calling algorithms have been developed to not only identify genome-wide enrichment of aligned reads, but also to distinguish peaks of modified histones from noise and artifacts. Model-based analysis of ChIP-Seq version 2 (MACS2), a widely-used peak calling algorithm for ChIP-seq, and other ChIP-seq peak calling methods are designed to address the high rate of background in ChIP-seq and are vulnerable to mistaking background signal as peaks particularly when the background is low [37–39]. Sparse Enrichment Analysis (SEACR) has been developed to perform peak calling from CUT&RUN data that, like CUT&Tag data, is characterized by low background. However, no peak calling algorithms have been designed to address both the low background and peak profile variability that is characteristic of histone modification CUT&Tag data [39].

Here, we present GoPeaks, a peak calling algorithm designed for histone modification CUT&Tag data. We compared the performance of GoPeaks against other widely used peak calling algorithms to detect H3K4me3, H3K4me1, H3K27me3, and H3K27ac peaks from CUT&Tag data. We demonstrate that GoPeaks robustly detects genome-wide histone modifications and notably, identifies H3K27ac with improved sensitivity compared to other peak callers. Moreover, we showed that GoPeaks can be used to detect transcription factor and chromatin accessibility peaks from ChIP-seq, CUT&RUN, and ATAC-seq data.

## Results

### Peak calling with a binomial distribution and a minimum count threshold

**Fig. 2:**
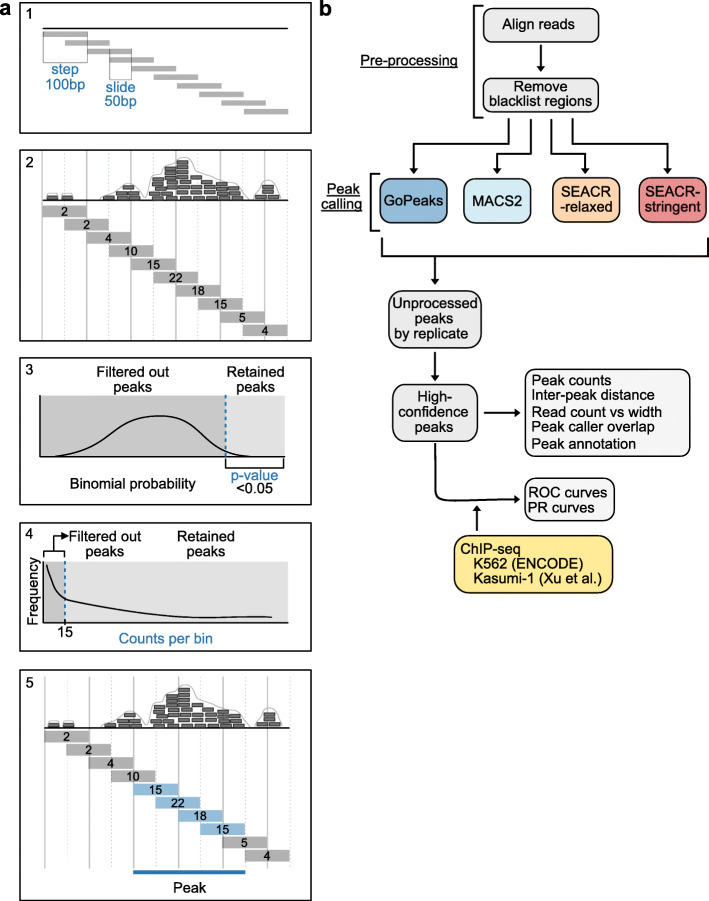
Overview of the GoPeaks methodology and benchmarking workflow. **a** Five general steps of the GoPeaks peak calling methodology. Each panel (a1-a5) represents a separate step. (a1) Step indicates the bin width and slide (the width of the bin overlap). (a2) Counting the number of aligned reads per bin. (a3) Example of a Binomial probability test distribution and threshold to retain significantly different peaks. (a4) Filtering out bins with less than 15 counts. (a5) Retained bins within 150 bp are merged and identified as a peak. **b** Schematic overview of the benchmarking workflow. All CUT&Tag datasets undergo the same pre-processing and are separately analyzed by the peak calling methods. The unprocessed peaks are extracted for sub-analyses. High-confidence peaks were defined by taking the union of statistically significant peaks from all replicates and retaining the peaks present in at least two biological replicates. Generation of the receiver operator characteristic (ROC) and precision-recall (PR) curves require ChIP-seq standards

GoPeaks performs genome-wide peak identification of histone modification binding from CUT&Tag data in five general steps (Fig. 2a). First, GoPeaks bins the genome into small intervals. Users can control the width of each bin with the “step” parameter and the width of bin overlap with the “slide” parameter. GoPeaks then quantifies the number of aligned reads contained within each bin. In bins greater than 15 counts (set by the “minreads” parameter), GoPeaks uses a Binomial distribution to determine whether the bin counts are significantly different from the genome-wide distribution of aligned reads. Bins with a significantly large number of counts are retained (*p* value less than 0.05 before Benjamini-Hochberg correction by default). Finally, significant bins are merged into peaks if they are located within 150 bp of each other, which can be adjusted with the “mdist” parameter. In contrast, MACS2 slides across the genome using an empirically-derived window size and deploys a dynamic Poisson distribution to evaluate the likelihood that aligned reads within a given window region are statistically significant [37]. Following peak *p*-value correction using the Benjamini-Hochberg procedure, MACS2 then merges overlapping significant regions into a peak. SEACR, on the other hand, bins the genome by regions with contiguous, non-zero signal blocks and calls regions with counts greater than an empirically-derived threshold based on the global distribution of background counts as peaks [39].

We developed a computational workflow to compare the performance of GoPeaks against MACS2 and SEACR to identify histone modification peaks from CUT&Tag data (Fig. 2b). We evaluated both SEACR threshold parameters, “SEACR-relaxed” and “SEACR-stringent”, as well as MACS2 “narrowPeaks” unless otherwise stated. A false discovery rate (FDR) threshold of 0.05 for GoPeaks and MACS2 as well as SEACR’s standard empirical FDR threshold was used [39]. We evaluated each peak callers’ ability to identify peaks from CUT&Tag sequencing using publicly available H3K4me1, H3K4me3, and H3K27me3 CUT&Tag data in K562 cells, a cell line model of blast-phase chronic myeloid leukemia (CML), and our H3K27ac CUT&Tag data in Kasumi-1 cells, an acute myeloid leukemia (AML) cell line. Each CUT&Tag dataset was aligned to the GRCh38 genome and the ENCODE blacklist regions were removed [40]. The unprocessed peaks and the high-confidence peaks, defined by taking the union of statistically significant peaks from all replicates and retaining the peaks present in at least two biological replicates, from each CUT&Tag dataset were used to quantify peak characteristics detected by each peak caller. We measured the sensitivity and specificity of each peak caller by their ability to recall peaks from publicly available ChIP-seq standards. We filtered the ChIP-seq standards for peaks with -log_10_(*p* value) > 10 and merged adjacent peaks within 1000 bp.

### Identification of narrow H3K4me3 peaks

**Fig. 3:**
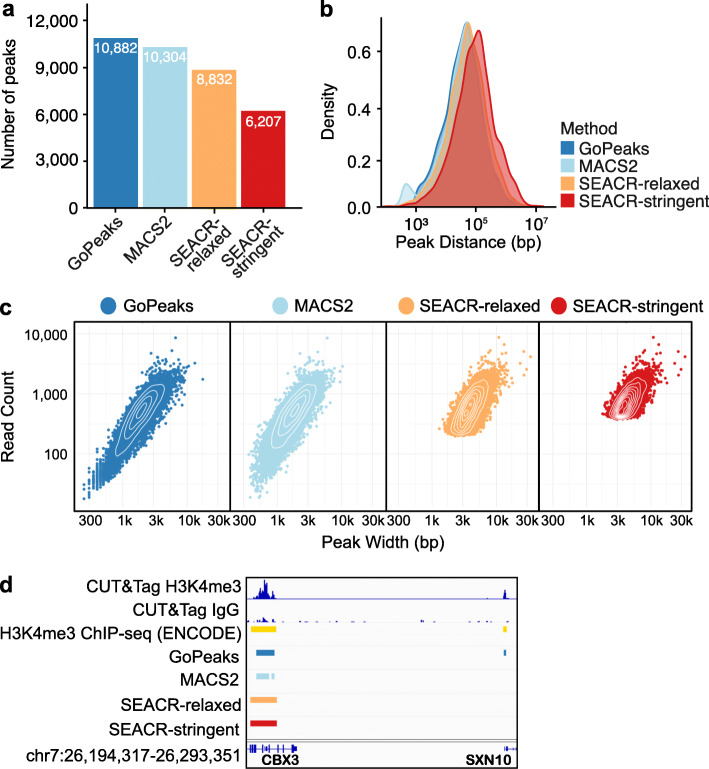
GoPeaks and MACS2 perform better than SEACR at identifying a range of H3K4me3 peak sizes. **a** Number of high-confidence peaks identified from H3K4me3 CUT&Tag data in K562 cells per peak calling method. Colors indicate the peak calling method. **b** Distribution of the distances to the next nearest peak. **c** Distribution of read counts by peak width. Each dot represents the read count and peak width of a single peak. **d** Example peaks at the *CBX3* and *SNX10* genes. IgG replicates are negative controls. Consensus peak calls for each method are shown. Tracks are CPM normalized and are scaled to the range [0–5.10] by IGV. Tracks are depicted on the GRCh38 genome assembly

We first compared the peak counts and characteristics detected by each peak calling algorithm from H3K4me3 CUT&Tag data. GoPeaks and MACS2 identified the greatest number of H3K4me3 peaks (Fig. 3a). To assess the characteristics of the peaks called by each algorithm, we calculated the average distance to the next nearest peak. This measurement indicates whether peak calling algorithms are splitting up enriched regions and inflating the peak count total. We found that the peaks called by GoPeaks were similar distances apart as MACS2 and SEACR-relaxed (Fig. 3b). MACS2, however, detected a small population of peaks less than 1000 bp apart. The peaks called by SEACR-stringent were noticeably farther apart than the other methods. To directly measure peak sizes identified by each peak calling method, we quantified the number of counts in each peak and the peak width. We found that GoPeaks and MACS2 called peaks across a range of widths (Fig. 3c). Both SEACR-relaxed and SEACR-stringent did not identify any peaks with a width less than 100 bp, potentially missing or aggregating important regions. As an example, all peak callers recognized a peak overlapping the *CBX3* and *HNRNPA2B1* promoters that is approximately 8500 bp wide (Fig. 3d). However, only GoPeaks identified a peak located at the promoter of *SNX10* nearly 1450 bp wide. Together, these results demonstrate GoPeaks’ ability to identify H3K4me3 peaks across a range of sizes.

### Sensitivity and specificity of detecting narrow H3K4me3 peaks

**Fig. 4:**
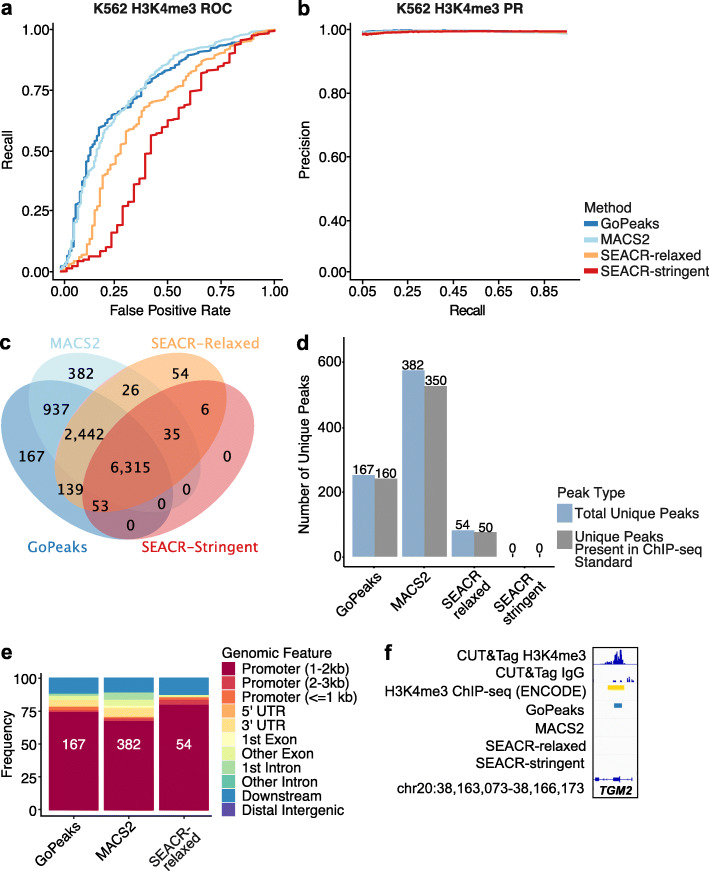
GoPeaks has a favorable specificity and sensitivity for narrow H3K4me3 CUT&Tag peaks. **a** ROC curves quantifying the recall and false positive rates and **b** PR curves quantifying the precision and recall rates of H3K4me3 CUT&Tag data from H3K4me3 ChIP-seq data. Both ChIP-seq and CUT&Tag datasets were generated in K562 cells. Colors indicate the peak calling method. **c** Overlap of high-confidence peaks identified by each peak caller. **d** Comparison of unique peaks that are identified by each peak calling algorithm and are also present in the ChIP-seq standard. Each bar is labeled by the number of peaks it represents. Colors indicate the peak type. **e** Annotation of unique peaks identified by each peak caller. Each bar is labeled by the number of unique peaks. Colors indicate the genomic feature. Downstream is at least 300 bp towards 3′ end of DNA strand. **f** Example peaks at the *TGM2* gene. IgG replicates are the negative controls. Consensus peak calls for each method are shown. Tracks are CPM normalized and are scaled to the range [0–1.46] by IGV. Tracks are depicted on the GRCh38 genome assembly. UTR, untranslated region

While both GoPeaks and MACS2 identify more H3K4me3 peaks than the SEACR peak calling methods, it is unclear whether some of these peaks may be false positives. To understand the sensitivity and specificity of each peak caller for H3K4me3 marks, we compared the peaks identified from publicly available K562 CUT&Tag data to those identified by ChIP-seq on the same cell line from the ENCODE Project [19, 41]. We created receiver operating characteristic (ROC) curves, which maps the true positive rate, or recall, against the false positive rate. A true positive is defined as a peak identified in the K562 CUT&Tag data, which is also present in the ENCODE K562 ChIP-seq data. ROC curves along with precision-recall (PR) curves, which instead quantify the relationship between precision and recall, were used to characterize peak caller sensitivity and specificity. GoPeaks and MACS2 demonstrated a greater degree of peak recall than the SEACR methods for a given false positive rate (Fig. 4a). GoPeaks and MACS2 had comparable area under the ROC curve (AUROC), which is a measurement of how well the peak callers detect CUT&Tag peaks that are also present in the ChIP-seq standard (Additional file 1: Fig. S1a). Every method demonstrated a similar ability to identify peaks with high precision across a range of recall values (Fig. 4b; Additional file 1: Fig. S1b). It is unlikely to observe perfect concordance due to the technical differences between the CUT&Tag and ChIP-seq assays. Indeed, given the sensitivity of CUT&Tag, it is likely that CUT&Tag will identify regions enriched with aligned reads that are not evident in ChIP-seq data.

GoPeaks’ high sensitivity and specificity is likely due in part to its ability to identify peaks not captured by the other peak calling algorithms. To assess what may have distinguished each peak callers’ performance, we studied the overlap of the high-confidence peaks. GoPeaks and MACS2 called the majority of peaks detected by both SEACR-stringent and SEACR-relaxed (Fig. 4c). In addition, GoPeaks identified 167 peaks not detected by any other peak caller, which likely contributed to GoPeaks’ high sensitivity and specificity as 95.8% (160) of GoPeaks’ unique peaks were also present in the ChIP-seq standard (Fig. 4d; Additional file 1: Fig. S1c). Since H3K4me3 peaks are associated with promoters, we annotated each peak set to the nearest genomic feature [5, 23, 24]. The unique peaks identified by GoPeaks, MACS2, and SEACR-stringent were mostly associated with promoters (77.8%, 70.2%, and 85.2%, respectively), consistent with the established biology of H3K4me3 (Fig. 4e; Additional file 2: Table S1). SEACR-stringent did not identify any unique peaks. Consistent with these findings, GoPeaks detected a peak in both CUT&Tag replicates located at the promoter of *TGM2* that was not identified by the other peak calling methods (Fig. 4f). Collectively, these results reveal that GoPeaks has favorable sensitivity and specificity for H3K4me3 data when compared with ChIP-seq, enabling the identification of an increased number of true positive peaks with a minimal false positive rate and at high precision.

### Sensitivity and specificity of detecting broad H3K4me1 peaks

**Fig. 5:**
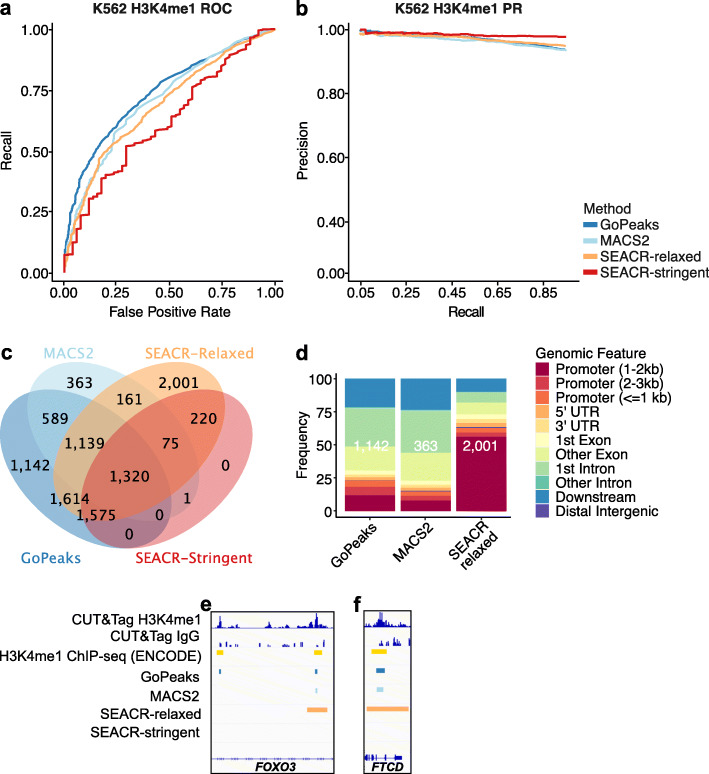
GoPeaks has a favorable specificity and sensitivity for broad H3K4me1 CUT&Tag peaks. **a** ROC curves quantifying the recall and false positive rates and **b** PR curves quantifying the precision and recall rates of H3K4me1 CUT&Tag data from H3K4me1 ChIP-seq data. Both ChIP-seq and CUT&Tag datasets were generated in K562 cells. Colors indicate the peak calling method. **c** Overlap of high-confidence peaks identified by each peak caller. **d** Annotation of unique peaks identified by each peak caller. Each bar is labeled by the number of unique peaks. Colors indicate the genomic feature. Downstream is at least 300 bp towards 3′ end of DNA strand. **e**, **f** Example peaks at the e. *FOXO3* gene and f. *FTCD* genes. IgG replicates are the negative controls. Consensus peak calls for each method are shown. Tracks are CPM normalized and are scaled to the range [0–1.31] for e. and [0–1.33] for f. by IGV. Tracks are depicted on the GRCh38 genome assembly. UTR, untranslated region

While GoPeaks was highly sensitive and specific to narrow H3K4me3 peaks, we wanted to evaluate its performance to detecting broad H3K4me1 peaks. To compare the performance of each peak caller to detect H3K4me1 CUT&Tag peaks from K562 cells, we measured their sensitivity and specificity against ENCODE H3K4me1 ChIP-seq on the same cell line [19, 41]. Overall, GoPeaks demonstrated comparable sensitivity and specificity across both H3K4me1 replicates (Fig. 5a, b; Additional file 1: Fig. S2a, b).

GoPeaks’ sensitivity and specificity may be due to its ability to detect H3K4me1 marks in intronic and intergenic regions. We therefore evaluated the overlap of the high-confidence peaks identified by each peak calling method. SEACR-relaxed identified the greatest number of unique H3K4me1 peaks among the four peak calling algorithms (Fig. 5c; Additional file 1: Fig. S2c). GoPeaks still identified 1142 unique peaks, 85.5% (976) of which were also present in the ChIP-seq standard (Additional file 1: Fig. S2d). To confirm the genomic features associated with the H3K4me1 peaks, we annotated each peak set to the nearest genomic feature. The unique peaks identified by GoPeaks and MACS2 were primarily associated with intronic and intergenic regions (76.8% and 85.1%, respectively) whereas SEACR-relaxed were mostly associated with promoters (62.7%; Fig. 5d; Additional file 2: Table S2). While H3K4me1 is found at active promoters, it displays the greatest enrichment at enhancers [5, 23, 24]. As an example, GoPeaks was able to identify a unique peak in the intronic region of *FOXO3* and the center of another H3K4me1 peak with greatest density of H3K4me1 signal (Fig. 5e). Both SEACR methods, on the other hand, called regions with widths greater than the region annotated by the ChIP-seq standard. In regions where the intronic region is much smaller, like in *FTCD*, resolving the center of peaks is crucial (Fig. 5f). Both SEACR methods identify genomic features that include the promoter, exonic, and intronic regions of the *FTCD* gene, in contrast to what is annotated in the ChIP-seq standard as well as detected by GoPeaks. Together, these findings reveal that GoPeaks has favorable sensitivity and specificity in identifying H3K4me1 peaks while simultaneously calling sufficiently narrow peaks to separate promoter and non-promoter regulatory regions.

### Sensitivity and specificity of detecting broad H3K27me3 peaks

Although we demonstrated that GoPeaks robustly detects peaks of histone modifications associated with euchromatic regions of the genome, we wanted to understand the ability of GoPeaks to identify H3K27me3 peaks, which are associated with regions of repressed gene transcription [5, 29–32]. Similar to the peak profiles of H3K4me1, H3K27me3 peaks tend to span broad regions. However, H3K27me3 signal can be detected across entire bodies of silence genes [5, 29–32]. To address this, we developed a “--broad” flag for GoPeaks to alter the width of genome bins to 5000 bp and bin overlap to 1000 bp, enabling the capture of peaks with broad domains. While MACS2 also has a broad peaks feature that increases the gap length to merge nearby enriched peaks, the SEACR methods do not [37, 39].

**Fig. 6:**
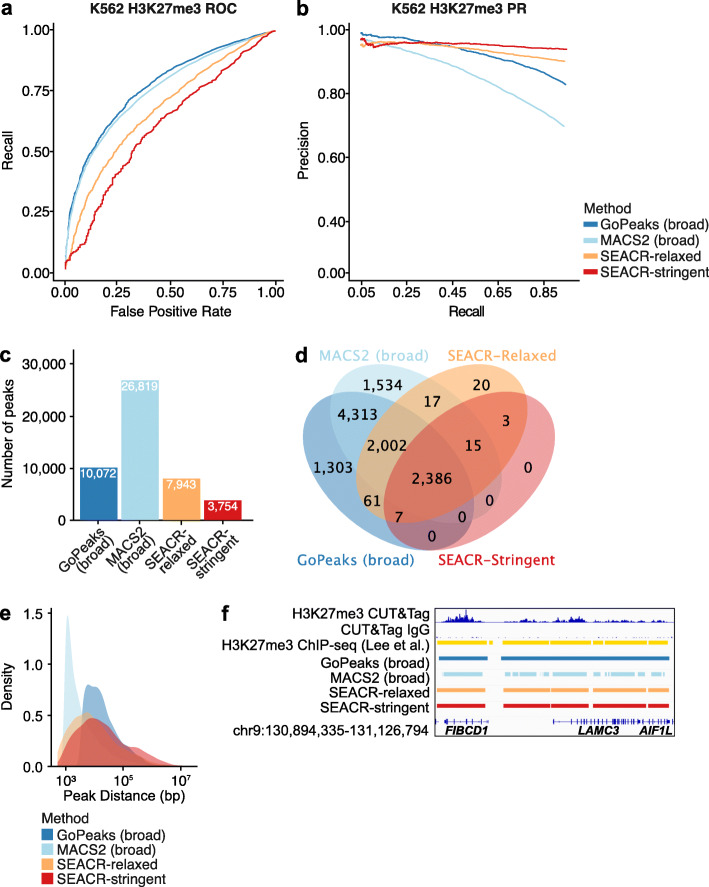
GoPeaks captures the broad peak profiles of H3K27me3 CUT&Tag peaks. **a** ROC curves quantifying the recall and false positive rates and **b** PR curves quantifying the precision and recall rates of H3K27me3 CUT&Tag data from H3K27me3 ChIP-seq data. Both ChIP-seq and CUT&Tag datasets were generated in K562 cells. Colors indicate the peak calling method. GoPeaks and MACS2 “--broad” flags were used. **c** Number of high-confidence peaks identified from H3K4me3 CUT&Tag data per peak calling method. **d** Overlap of high-confidence peaks identified by each peak caller. **e** Distribution of the distances to the next nearest peak. **f** Example peaks at *FIBCD1*, *LAMC3*, and *AIF1L* genes. IgG replicates are the negative controls. Consensus peak calls for each method are shown. Tracks are CPM normalized and are scaled to the range [0–18.0] by IGV. Tracks are depicted on the GRCh38 genome assembly

We compared the sensitivity and specificity of the GoPeaks and MACS2 broad methods and the standard SEACR methods to detect H3K27me3 CUT&Tag peaks from K562 cells, using ENCODE H3K27me3 ChIP-seq data from the same cell line as the standard [19, 41]. While GoPeaks and MACS2 had enhanced AUROC as compared to the SEACR methods, MACS2 had a considerably lower AUPRC (Fig. 6a, b; Additional file 1: Fig. S3a, b). As precision is dependent on the number of true positive peaks, we measured the number of peaks identified by each method. MACS2 detected 26,819 peaks, more than double the peak count of the next closest method (Fig. 6c). However, 94.3% (25,285) of these peaks overlapped peaks identified by the other methods (Fig. 6d; Additional file 1: Fig. S3c). Moreover, of the 1534 peaks uniquely identified by MACS2, only 38.6% (592) were present in the standard (Additional file 1: Fig. S3d). In contrast, GoPeaks detected a comparable number of unique peaks (1303), the majority of which were present in the standard (60.9%; 793). We hypothesized that MACS2 may be splitting the broad domains of enriched H3K27me3 signal into small peaks, so we measured the distribution of peak sizes and distances to the next nearest peak. Indeed, we identified a large population of MACS2 peaks that contained less than 100 counts (Additional file 1: Fig. S3e) and that MACS2 peaks were substantially closer together than the other methods (Fig. 6e). As an example, GoPeaks and the SEACR methods preserved the broad domain of enriched H3K27me3 signal spanning the gene bodies of *FIBCD1*, *LAMC3*, and *AIF1L* (Fig. 6f). Even with the “--broad” flag, MACS2 breaks up these domains into small peaks. These findings demonstrate that GoPeaks detects broad domain peaks within heterochromatic regions with high sensitivity and specificity.

### Sensitivity and specificity of detecting broad and narrow H3K27ac peaks

**Fig. 7:**
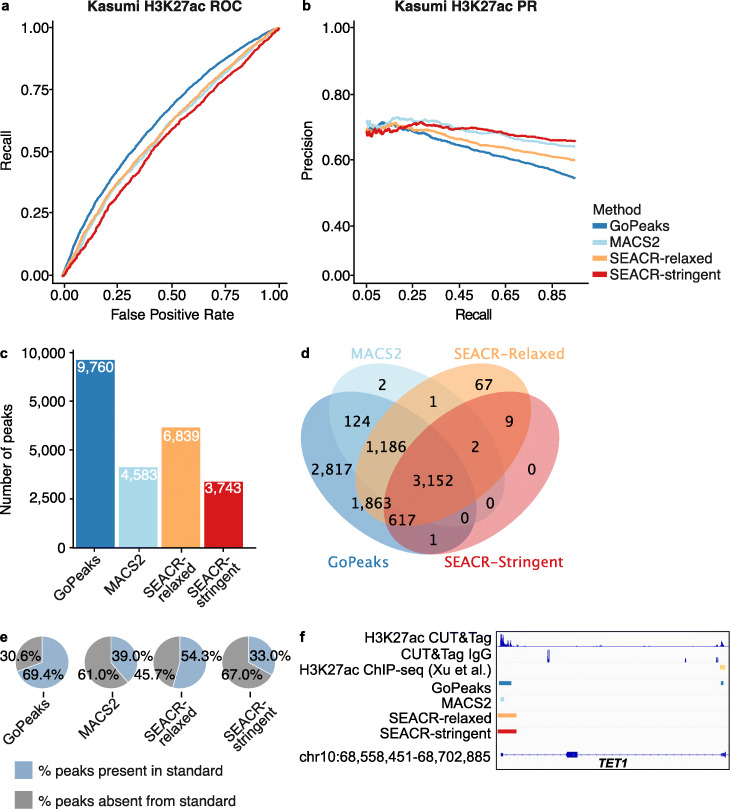
GoPeaks has higher specificity and sensitivity for H3K27ac CUT&Tag peaks with broad and narrow peak shapes. **a** ROC curves quantifying the recall and false positive rates and **b** PR curves quantifying the precision and recall rates of H3K27ac CUT&Tag data from H3K27ac ChIP-seq data. Both ChIP-seq and CUT&Tag datasets were generated in Kasumi-1 cells. Colors indicate the peak calling method. **c** Number of high-confidence peaks identified from H3K4me3 CUT&Tag data per peak calling method. **d** Overlap of high-confidence peaks identified by each peak caller. **e** Percent of total H3K27ac ChIP-seq standard peaks that are identified by each peak caller. **f** Example peaks near at the *TET1* gene. IgG replicates are the negative controls. Consensus peak calls for each method are shown. Tracks are CPM normalized and are scaled to the range [0–4.26] by IGV. Tracks are depicted on the GRCh38 genome assembly

H3K27ac marks are crucial for defining active genomic features and can have characteristics of broad and narrow peaks [27, 28]. To evaluate the performance of GoPeaks on H3K27ac, we performed CUT&Tag sequencing for H3K27ac on Kasumi-1 cells. We again measured the number and characteristic of peaks called in CUT&Tag data as compared to ChIP-seq. Since ENCODE does not have H3K27ac ChIP-seq data for Kasumi-1 cells, we identified the high-confidence peaks of published H3K27ac ChIP-seq data on the same cell line [42]. While GoPeaks showed an enhanced ability to recall peaks across a range of false positive rates, this may have been at the expense of its precision and recall (Fig. 7a, b; Additional file 1: Fig. S4a, b). Since precision is dependent on the number of true positives identified by each caller, we once again measured the number of peaks identified by each method. Indeed, GoPeaks detected a total of 9760 high-confidence peaks, which is nearly 3000 more peaks than what was detected by the closest peak caller, as well as 2817 peaks that were not detected by any other peak caller (Fig. 7c, d; Additional file 1: Fig. S4c). While only 1078 peaks of the unique peaks were present in the standard (Additional file 1: Fig. S4d), GoPeaks identified 69.4% of all peaks present in the ChIP-seq standard (Fig. 7e). In contrast, SEACR-stringent, which demonstrated improved precision and recall over the other peak calling methods, identified the least number of total peaks (3743). Moreover, SEACR-stringent did not detect any unique peaks and only identified 33.0% of the peaks present in the standard. Overall, GoPeaks identified a substantial number of H3K27ac peaks that were present in the ChIP-seq standard with some trade-off to its precision.

GoPeaks identified peaks across a range of widths, which is crucial for H3K27ac peak detection (Additional file 1: Fig. S4e). As an example, GoPeaks identified a peak with both narrow and broad characteristics at the promoter of *TET1* (Fig. 7f). While GoPeaks and SEACR identified the whole peak, MACS2 only identified the narrow portion of the peak. Additionally, GoPeaks identified another narrow peak in an exonic region of *TET1*, which is also present in the ChIP-seq standard. Together, this data highlights GoPeaks’ dynamic range to identify both the narrow and broad peaks, which are characteristic of H3K27ac marks.

### Identification of transcription factor and chromatin accessibility peaks using GoPeaks

**Fig. 8:**
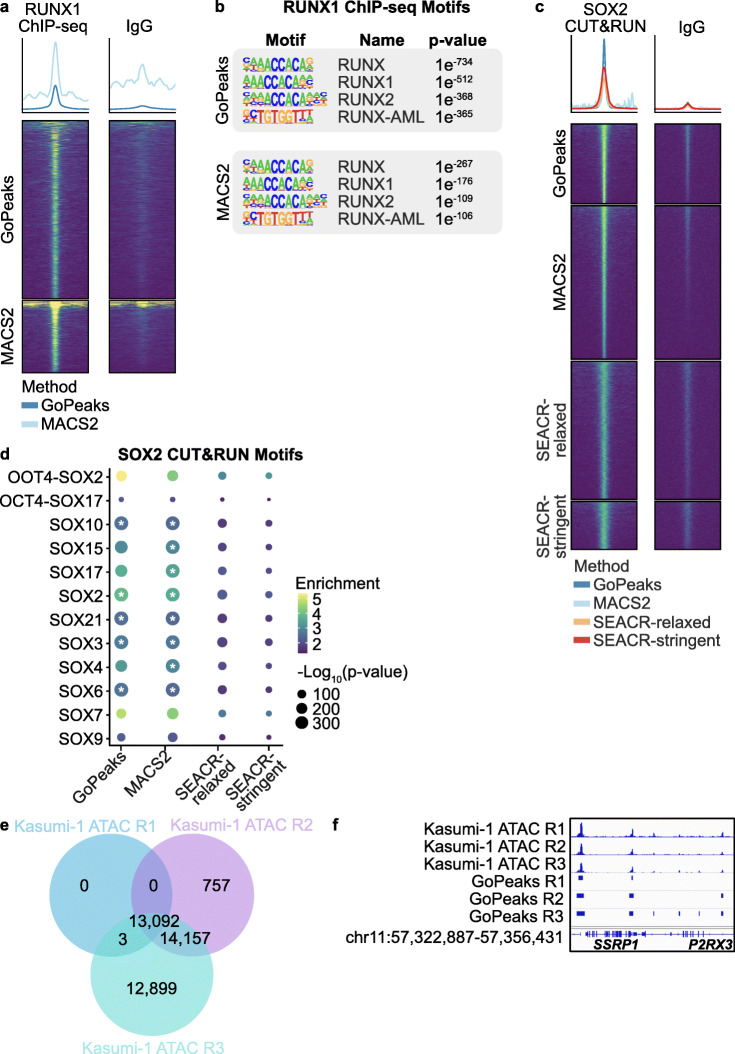
GoPeaks detects peaks from other epigenetic profiling techniques. **a** Heatmap of global RUNX1 and IgG ChIP-seq signal from K562 cells per peak calling method. **b** Transcription factor motif enrichment for regions of global RUNX1 ChIP-seq signal. Top four known motifs are shown. **c** Heatmap of global SOX2 and IgG CUT&RUN signal from H1 hESC cells per peak calling method. **d** Transcription factor motif enrichment for regions of global SOX2 CUT&RUN signal. Dot color represents the binomial fold enrichment and color represents the -log_10_(*p-*value) of the motif. The asterisk indicates the motif *p*-value is less than R’s smallest representable number (1 × 10^−324^). **e** ATAC-seq was performed on Kasumi-1 cells. Overlap of peaks identified in each biological replicate by GoPeaks. **f** Example peaks at *SSRP1* and *P2RX3* genes. Peak calls for individual biological replicates are shown. Tracks are CPM normalized and are scaled to the range [0–3.11] by IGV. Tracks are depicted on the GRCh38 genome assembly

While GoPeaks was designed for histone modification CUT&Tag data, the GoPeaks peak calling methodology can be applied to any epigenetic profiling data with read pileups aligned to a genome. Therefore, we wanted to determine the applicability of GoPeaks to other epigenetic profiling techniques, including ChIP-seq, CUT&RUN, and Assay for Transposase-Accessible Chromatin with sequencing (ATAC-seq). Moreover, surveying these methods allowed us to understand whether GoPeaks is capable of identifying peaks associated with transcription factors and chromatin accessibility. We first assessed the ability of GoPeaks to detect transcription factor binding events from RUNX1 ChIP-seq from K562 cells and SOX2 CUT&RUN from H1 human embryonic stem cells (hESC) [41, 43]. For the analysis of ChIP-seq data, we compared the characteristics of peaks detected by GoPeaks only to MACS2 as SEACR was developed for CUT&RUN data [39]. We found that both GoPeaks and MACS2 detected peaks from the K562 RUNX1 ChIP-seq data that were enriched for RUNX transcription factor binding motifs (Fig. 8a, b) [44]. Moreover, both GoPeaks and MACS2 detected the enrichment of SOX bindings motifs with high confidence from the H1 hESC SOX2 CUT&RUN data (Fig. 8c, d) [44]. These findings show that GoPeaks can identify transcription factor binding events from ChIP-seq and CUT&RUN data. To understand whether GoPeaks detects chromatin accessibility peaks, we performed ATAC-seq in Kasumi-1 cells. GoPeaks identified 13,092 overlapping peaks in the three biological replicates (Fig. 8e). GoPeaks detected the most peaks in the R3 biological replicate with an additional 14,157 peaks that were shared by the R2 replicate as well as 12,899 peaks only present in the R3 replicate. This was evident at the *SSRP1* and *P2RX3* genes where GoPeaks identified enriched regions of chromatin accessibility in all three replicates, but also detected additional low count peaks in the R3 replicate (Fig. 8f). Collectively, these findings demonstrate the applicability of GoPeaks to call transcription factor and chromatin accessibility peaks.

## Discussion

GoPeaks was designed to address the low background and peak profile variability characteristic of histone modification CUT&Tag data. GoPeaks demonstrated a favorable ability to detect peaks and were highly sensitive and specific to calling peaks across a range of histone modification CUT&Tag data. These results were particularly encouraging for H3K27ac peaks, which exhibit both narrow and broad characteristics. Since H3K27ac is a marker of active promoters and enhancers, it is crucial to pinpoint active regulatory non-coding elements. Moreover, we demonstrated that GoPeaks can identify transcription factor and chromatin accessibility peaks from ChIP-seq, CUT&RUN, and ATAC-seq data.

MACS2 and SEACR both demonstrated biases towards the identification of narrow or broad peaks, respectively. MACS2 performed particularly well in analyzing H3K4me3 CUT&Tag data, in which peaks tend to be sharply localized. MACS2 identified a comparable amount of H3K4me3 CUT&Tag peaks as GoPeaks with similar sensitivity and specificity. The bias of MACS2 for narrow peaks was also evident when calling peaks from H3K27me3 CUT&Tag data. Even with the MACS2 broad feature, MACS2 fragmented enriched H3K27me3 domains that spanned the entire gene bodies into small peaks. As MACS2 was designed to identify narrow transcription factor peaks in ChIP-seq data, its bias for narrow peaks is unsurprising. SEACR, in contrast, demonstrated a favorable ability in detecting broad H3K4me1 peaks [38]. SEACR-relaxed identified the most unique H3K4me1 peaks, but with comparable precision and recall to GoPeaks. The reason for SEACR’s bias for broad marks may be due to its empirical segmentation of the genome with contiguous, non-zero signal blocks [39]. Since the signal blocks are not fixed, peaks may contain excess regions with low counts that are adjacent to a true peak (Fig. 5f). GoPeaks avoids this potential problem as each bin has a fixed width and is evaluated for significance before merging. GoPeaks’ simple but flexible framework is more amendable to the identification of both broad and narrow peaks, like those present in H3K27ac data.

SEACR was more conservative in the identification of peaks across all marks. SEACR-stringent, in particular, consistently detected less peaks than the other methods. This strategy seems to be beneficial for SEACR’s precision and recall, notably in the detection of H3K27ac peaks, and may be more appropriate for researchers that have a low threshold for calling false positives. GoPeaks, on the other hand, may be best suited for researchers that are interested in discovering new peaks with a small trade-off in precision. However, GoPeaks largely performed at comparable precision and improved recall over the other peak callers. Our analysis demonstrates GoPeaks detects a substantial number of histone modification peaks at high sensitivity and specificity.

There are important limitations to consider in this analysis. We only evaluated the sensitivity and specificity of each peak calling method using four CUT&Tag histone modification datasets. Although the modifications studied are likely important for epigenetic studies, the peak profiles cover a broad range that will be likely encountered by other marks. We encourage users to test GoPeaks on other histone modification datasets as well as transcription factor and chromatin accessibility datasets. While we demonstrated that GoPeaks can call peaks from these modalities, more work is needed to characterize its performance. Additionally, we only tested these peak calling methods in three different cell lines. We cannot confidently rule out that GoPeaks may have a biological bias for K562, Kasumi-1, or H1 hESC although this is unlikely. Lastly, there were no CUT&Tag standards for the ROC studies. Although it would have been preferable to compare peaks between CUT&Tag datasets, the CUT&Tag technique is still new and few datasets exist in the public domain. While ChIP-seq served as a useful comparator given the abundance of publicly available data, the fundamental differences in the ChIP-seq and CUT&Tag methods as well as the histone modification antibodies used to generate each dataset should be considered when comparing the results of these two methods. Notably, the antibodies used for the H3K4me1 and H3K27me3 CUT&Tag and ChIP-seq experiments were the same, but the antibodies for H3K4me3 and H3K27ac were not. However, our analysis indicates GoPeaks’ ability to extract biological meaning from CUT&Tag data. Overall, GoPeaks demonstrated to be a robust peak calling method across a range of histone modification CUT&Tag data.

## Conclusions

GoPeaks is a peak calling algorithm designed for histone modification CUT&Tag data. We showed that GoPeaks detects peaks of histone modifications that are frequently used in epigenetic studies with high sensitivity and specificity. Notably, GoPeaks demonstrated an improved ability to identify H3K27ac peaks, which are critical to localizing active regulatory non-coding elements throughout the genome, over other standard peak calling algorithms. Moreover, we showed that GoPeaks is able to detect peaks from other epigenetic profiling techniques, including ChIP-seq, CUT&RUN, and ATAC-seq.

## Methods

### GoPeaks algorithm

GoPeaks detects peaks from aligned, paired-end sequencing reads by calculating read coverage in genome bins (“step” 100 bp and “slide” 50 bp by default). Reads from each sample are normalized to counts per million (CPM) and, when a negative control experiment is provided (e.g., IgG, input), then scaled per pin using a custom scaling function:

$$ {\mathrm{sample}}_{\mathrm{bin}}=\left\{\begin{array}{c}{\mathrm{sample}}_{\mathrm{bin}}>{\mathrm{negativeControl}}_{\mathrm{bin}},\kern0.5em {\mathrm{sample}}_{\mathrm{bin}}\times \left(1-\frac{\mathrm{CPM}\left({\mathrm{negativeControl}}_{\mathrm{bin}}\right)}{\mathrm{CPM}\left({\mathrm{sample}}_{\mathrm{bin}}\right)}\right)\\ {}{\mathrm{sample}}_{\mathrm{bin}}<{\mathrm{negativeControl}}_{\mathrm{bin}},\kern0.5em 0\end{array}\right. $$

GoPeaks then collects important parameters “*n*” and “*p*” to model the Binomial distribution. “*n*” is equal to the number of reads and “*p*” represents the probability of success, where success is defined as choosing “*x*” reads in a genome bin from “*n.*” “*p*” is estimated as the average read depth in all non-zero coverage bins over the number of non-zero bins, divided by “n”. Modeling read counts using a Binomial distribution was originally inspired by works from the Regulatory Genomics Toolbox [45]. GoPeaks then traverses the genome and tests bins greater than “minreads” (15 by default), followed by multiple hypothesis correction using Benjamini-Hochberg. Bins are filtered by their adjusted *p*-values with “-p”. Adjacent bins are merged with the “--mdist” parameter (150 bp by default) into peaks, where they are filtered using a minimum peak width flag with “--minwidth” (150 bp by default).

#### Method comparison workflow

##### Pre-processing

K562 H3K4me3 (GEO accession GSM3536516), H3K4me1 (GEO accession GSM3536518), H3K27me3 (GEO accession GSM3560261), and IgG (GEO accession GSM3560264) CUT&Tag data from Kaya-Okur et al. 2019 as well as H1 hESC SOX2 (GEO accession SRR8748855 and SRR8748856) and IgG (GEO accession SRR8748845) CUT&RUN data from Meers et al. 2019 were downloaded through National Center for Biotechnology Information Gene Expression Omnibus (NCBI GEO) [19, 43, 46]. K562 RUNX1 (ENCODE ID ENCSR414TYY) and the IgG input control (ENCODE ID ENCSR173USI) ChIP-seq data were downloaded from the ENCODE portal [41, 47]. All data were aligned to the GRCh38 genome with Bowtie2 with the following options “--local --very-sensitive-local --no-unal --no-mixed --no-discordant --phred33 -I 10 -X 700” [74].

##### Peak calling

GoPeaks (v1.0.0) used the optional flag “--mdist 1000” to merge peaks within 1000 bp. For H3K27me3 CUT&Tag, the flags “--mdist 3000” and “--broad” were used to adjust the step to 5000 bp and the slide to 1000 bp. MACS2 (v 2.2.6) used the “--format BAMPE” flag with a genome size of 2.7e9 and the standard FDR threshold of 0.05 [37]. MACS2 “--broad” was used for H3K27me3 CUT&Tag data and “narrowPeak” was used for all other data. SEACR (v 1.4) used the “norm” flag when treatment and IgG samples were used in addition to using the relaxed and stringent mode [39]. SEACR uses an empirical false discovery rate (FDR) calculated by quantifying the percentage of control signal blocks remaining out of the total above the threshold [39].

##### Post-processing

After peaks were called for each method, high-confidence peaks were defined by taking the union of statistically significant peaks from all replicates and retaining the peaks present in at least two biological replicates within a study’s data set via a custom script. The purpose of finding high-confidence peaks is to reduce spurious peaks called in only one replicate and focus on peaks that consistently appear in multiple replicates. Intervene was used on the high-confidence peak sets to find common and exclusive peaks across peak callers [48].

##### Peak characterization

Peak counting was done in base R. ChIPseeker was used to annotate peaks to the nearest transcription start site [49]. The read count at high-confidence peak intervals was tallied with BEDtools intersect –C to yield read depth density distributions, and peak-peak distances were calculated with GRanges [50, 51]. Enrichment of transcription factor motifs in RUNX1 ChIP-seq and SOX2 CUT&RUN peaks were identified using HOMER [44]. Data cleaning and visualization were mainly facilitated using data.table, ggplot2, and deeptools [52, 53]. Tracks were normalized by CPM and visualized using Integrative Genomics Viewer (IGV) [54].

##### Receiver operating characteristic and precision-recall curves

In the receiver operating characteristic (ROC) and precision-recall (PR) analyses, the ranking metrics for each peak calling algorithm was counts at high-confidence peaks (obtained through BEDtools intersect -C). The outputs of MACS2, SEACR, and GoPeaks high-confidence peak counts were the input for ROC and PR analyses. The high-confidence counts from CUT&Tag data were compared to publicly available ChIP-seq standards downloaded from the ENCODE portal and ChIP-Atlas [41, 47, 55]. K562 H3K4me3 (ENCODE ID ENCFF885FQN), H3K4me1 (ENCODE ID ENCFF759NWD), and H3K27me3 (ENCODE ID ENCFF795ZOS) ChIP-seq data was accessed from the ENCODE portal [41, 47]. Kasumi-1 H3K27ac (ChIP-Atlas SRX ID SRX4143063 and SRX4143067) ChIP-seq data was accessed from ChIP-Atlas [42, 55]. The standards were filtered for peaks with -log_10_(*p* value) > 10 and adjacent peaks were merged if they were within 1000 bp.

Custom scripts were used to threshold over unique values of the ranking metric to define predicted truth and false, which were intersected with the ChIP-seq standards to fill out the confusion matrix. True negatives are defined as peaks that did not meet the threshold for significance and were not annotated in the ChIP-seq standard. Secondary properties such as precision, recall, and FPR, were calculated from the primary properties. ROC curves were made by plotting precision versus false positive rate; PR curves were made by plotting precision versus recall. The area under the AUROC and AUPR curves were approximated with Riemann Sums using trapezoids.

#### Cell culture and CUT&Tag methods

##### Cell lines

Kasumi-1 cells (ATCC) were cultured in RPMI (Gibco) supplemented with 20% fetal calf serum (FCS, HyClone), 2 mM GlutaMAX (Gibco), 100 units/mL penicillin, and 100 μg/mL streptomycin (Gibco). Cells were cultured at 5% CO_2_ and 37 °C. Cell lines were tested monthly for mycoplasma contamination.

##### CUT&Tag

Benchtop CUT&Tag was performed as previously described^8^. In brief, Kasumi-1 cells were counted, harvested, and centrifuged for 5 min at 300×g at room temperature. Cells were washed two times in 1.5 mL wash buffer (20 mM HEPES pH 7.5, 150 mM NaCl, 0.5 mM Spermidine, 1× Protease inhibitor cocktail). Concanavalin A magnetic coated beads (Bangs Laboratories) were activated in binding buffer by washing two times (20 mM HEPES pH 7.5, 10 mM KCl, 1 mM CaCl_2_, 1 mM MnCl_2_). Washed cells were separated into 100,000 cell aliquots and 10 ul of activated beads were added to each sample. Samples rotated at room temperature end over end for 7 min. Beads were separated with a magnetic and supernatant was removed. 1 μl of primary antibody was diluted 1:50 in antibody buffer (20 mM HEPES pH 7.5, 150 mM NaCl, 0.5 mM Spermidine, 1× Protease inhibitor cocktail, 0.05% digitonin, 2 mM EDTA, 0.1% BSA). The primary antibodies used were: H3K27ac (ab4729, Abcam) and Normal Rabbit IgG (#2729, CST). Cells were incubated overnight at 4 °C on a nutator. Primary antibody was replaced with a guinea-pig anti-rabbit secondary antibody (Antibodies Online, cat. no. ABIN101961) diluted to 1:100 in wash buffer. Samples were incubated for 45 min at room temperature on nutator. Secondary antibody was removed, and samples were washed 2X in dig-wash buffer (20 mM HEPES pH 7.5, 150 mM NaCl, 0.5 mM Spermidine, 1× Protease inhibitor cocktail, 0.05% Digitonin). pA-Tn5 transposase, prepared and loaded with adaptors as previously described [19], was diluted 1:250 in dig-300 buffer (20 mM HEPES pH 7.5, 300 mM NaCl, 0.5 mM Spermidine, 1× Protease inhibitor cocktail, 0.01% digitonin) and added to samples. Samples were incubated for 1 h at room temperature on nutator. Samples were washed 2X with dig-300 buffer then resuspended in tagmentation buffer (dig-300 buffer with 10 mM MgCl_2_). Samples were incubated at 37 °C for 1 h. DNA was extracted with a DNA Clean & Concentrator-5 kit (ZYMO). Samples were amplified by PCR using custom Nextera primers at 400 nM and NEBNext HiFi 2x PCR Master Mix (New England Biolabs) [56]. PCR conditions were set to 72 °C for 5 min, 98 °C for 30 seconds, 14-27 cycles of 98 °C for 10 sec, 63 °C for 10 sec, and 72 °C for 1 min. Libraries were purified with AMPure Beads (Beckman) and sequenced on a NextSeq 500 sequencer (Illumina) using 37 BP PE sequencing by Massive Parallel Sequencing Shared Resource at Oregon Health and Science University.

##### ATAC-seq

Samples were prepared as previously described [57]. In brief, cells were resuspended tagmentation master mix (25 ul of 2× tagmentation buffer, 2.5 ul of TDE1 [Illumina], 0.5 ul of 1% digitonin; 2× tagmentation buffer: 66 mM Tris-Acetate, pH 7.8, 132 mM potassium acetate, 20 mM magnesium acetate, 32% v/v N,N-Dimethylformamide). Samples were incubated at 37 °C for 30 min. DNA was purified using Zymo Clean and Concentrator 5 Kit (Zymo). Transposed DNA was amplified and purified as described previously with adapted primers [58, 59]. Samples were quantified using Qubit dsDNA HS Assay Kit (Invitrogen), pooled, and sequenced by Genewiz with a HiSeq-X (Illumina) using 75 BP PE sequencing.

## Supplementary Information

**Additional file 1: Supplementary Figs. S1-4.**

**Additional file 2: Supplementary Tables S1-2.**

**Additional file 3.** Review history.

## Data Availability

GoPeaks is free to use and is publicly accessible on GitHub (https://github.com/maxsonBraunLab/gopeaks) [60]. A stable version of the GitHub repository is available through Zenodo (https://zenodo.org/record/6413077#.YkydK5PMKAl) [61]. Custom scripts used to compare the peak calling algorithms are available in the gopeaks-compare repository (https://github.com/maxsonBraunLab/gopeaks-compare) [62]. The raw and processed sequencing datasets generated during the current study are available in the NCBI Gene Expression Omnibus (GEO) under accession number GSE190793 [46, 63]. The K562 H3K4me3 (GEO accession GSM3536516), H3K4me1 (GEO accession GSM3536518), H3K27me3 (GEO accession GSM3560261), and IgG (GEO accession GSM3560264) CUT&Tag datasets from Kaya-Okur et al. 2019 analyzed during the current study are available in the NCBI GEO [19, 46, 64–67]. The K562 H3K4me3 (ENCODE ID ENCFF246IEW), H3K4me1 (ENCODE ID ENCFF590NGQ), and H3K27me3 (ENCODE ID ENCFF795ZOS) ChIP-seq datasets analyzed during the current study are available in ENCODE [41, 47, 68–71]. The Kasumi-1 H3K27ac (ChIP-Atlas SRX ID SRX4143063 and SRX4143067) ChIP-seq data analyzed during the current study is available in ChIP-Atlas [42, 55, 72, 73].
