# Supplementary material for: GoPeaks: histone modification peak calling for CUT&Tag
Source: Genome Biol. 2022 Jul 4;23:144. doi: 10.1186/s13059-022-02707-w (PMC9252088; doi:10.1186/s13059-022-02707-w)
Supplement: Supplementary file 1 — Additional file 1: Supplementary Figs. S1-4. [file 13059_2022_2707_MOESM1_ESM.docx]

**1^st^ round**

**Reviewer 1**

In this manuscript, Yashar et al develop GoPeaks, a new peak calling tool for calling peaks from histone modification CUT&Tag datasets. There is a need for this type of peak calling tool, and therefore the authors present a compelling manuscript with data to match their claims. I have the following comments:

1. Did the authors try to use GoPeaks to call peaks from either ChIP-seq or CUT&RUN datasets? I think it is worthwhile to explore whether it can be applied to these other related techniques.

2. Related, did the authors try to use GoPeaks to call peaks from non-histone modification CUT&Tag (or related) datasets. There is plenty of available CUT&Tag (and other) data for factors such as RNAPII and others. It would be beneficial to the community to report whether GoPeaks can be used for these other datasets.

3. The authors do a good job with their introduction, although I find many citations missing. As a quick example, in line 73, the authors only cite low input CUT&Tag, when they also mention CUT&RUN, not citing either original (Skene et al 2017) or low input (Skene et al 2018, Hainer et al 2019) CUT&RUN

4. It is unclear whether negative control experiments (IgG) are taken into account in GoPeaks analysis.

5. The authors describe GoPeaks in the first paragraph of the results section. Since the authors then go on to compare GoPeaks performance to MACS2 and SEACR. It would be beneficial to the reader to quickly describe the peak identification process for MACS2 and SEACR for direct comparison.

6. The authors do a nice job comparing CUT&Tag data to ChIPseq data, such as when described on line 162. However, were the antibodies used the same? This might be important in the experimental data gathered and therefore when comparing peaks from these experiments.

7. The authors demonstrate robust results for H3K4me1, H3K4me3, and H3K27ac CUT&Tag data; however these are all euchromatic marks. Does GoPeaks work equally well (or superior to MACS and SEACR) for heterochromatic marks (such as H3K27me3 and H3K9me3)?

8. Although certainly up to the authors decision, a web-based served for GoPeaks would broaden appeal and usage by experimentalists, such as what was put together for SEACR.

9. There are a few minor typos, such as on line 136 "H3K3me3"

**Reviewer 2**

Cut&Tag is a recently developed sequence enrichment assay that offers improved signal to noise over ChIP-seq and requires less input material compared to Cut&Run. However, a peak calling tool specifically for use with Cut&Tag data is not currently available. Here authors report the development of a peak calling method termed 'GoPeaks' that is designed for use with Cut&Tag data. GoPeaks utilizes binomial distribution to determine if counts that map within genomic bins are significantly different from the genome-wide distribution of aligned reads. Using both narrow and broad histone modification datasets, and evaluating against ENCODE ChIP-seq data, GoPeaks performs comparatively well compared to existing peak calling tools (MACS2 and SEACR).

There is a need for new methods to analyze Cut&Tag data, and in addition depending on the mark, it can be challenging to identify an appropriate method to use. This manuscript offers a good description and assessment for new peak calling software for Cut&Tag data. I have several general critiques, as well as many minor edits to the article.

Critiques:

In our experience, Cut&Run and Cut&Tag frequently result in a set of background peaks that appear in IgG controls that should be subtracted from final peak sets (like blacklist sites). This manuscript lacks any discussion or information about such controls (with the exception of Figure 3 tracks and legend, but insufficient - was the CBX3 a called peak in IgG?).

It would be useful to provide a brief description of GoPeaks application with other types of data including Cut&Run and ATAC-seq data.

Consistently authors refer to high-confidence peaks as being identified in two biological replicates. First, it isn't clear that these peaks are derived from the intersection or union of the replicates, and second a measure of confidence is generally provided by using the Irreproducibility Discovery Rate (IDR) framework. Authors should clarify the use of replicates to derive 'high-confidence' peak sets and should really consider providing a more robust measure of what is termed high-confidence (i.e. IDR analysis). Similarly, authors also refer to peaks as high-quality peaks (e.g. line 247). This is not clear nor substantiated.

The sets of unique peaks identified in Figure 4c should be plotted via signal heatmaps and compared IgG to validate their signal and background.

Line 404, how much antibody was used?

Line 406, wash buffer references a company but lacks information.

Line 417, 14-27 cycles seems really high.

Figure 4e legend should remove or replace the term regulatory element and put a percentage sign within the barplot

Line 200, when comparing H3K4me1 peaks to ENCODE data clarify if broad or narrowpeaks were used.

Figure 1 represents H3K4me1 enrichment at intergenic regions, however, as discussed in the manuscript, this mark is present in both promoter and distal regions. Similarly, the Figure 1 legend lacks some information that describes elements of figure (i.e. black box).

Figure 2 legend mentions subfigure, would be better to use the term box or panel.

Figure 3d gene names should be italicized.

H3K27Ac should be written as H3K27ac, and H3K4Me1, H3K4Me3 (i.e. line 192) should be consistently written as H3K4me1 and H3K4me3.

Introduction paragraph 1 should consider using a single unified description of posttranslational histone modifications, as 3 different description/terms are used: chemical modifications, post-translation modifications and histone modifications.

Line 59 should be nucleosomes not histones.

Line 61 states 'binding of these factors' but it isn't clear what is being bound. Please rewrite sentence.

Lines 68 and 296 could also include analysis of other non-histone proteins such as TFs and co-regulators.

Line 69 implies that ChIP-seq always has high background. However, this is not true.

Line 139 poorly written statement. Also, would be good to provide brief description of the parameters for comparison (i.e., were they the same thresholds?).

Line 142-143, sentence confusing. I would state splitting up enriched regions over splitting up peaks into smaller peaks.

Line 145 to be consistent and clear 10^3 be should be written as 1,000 bp

Line 151 should read promoters that were approximately

Lines 152-153 and other instances (i.e., Lines 190-191, 216-218, etc.) authors discuss and reference biological function of individual genes. However, this is out of context and shouldn't be included in the results. I understand the point is to not miss important peaks, but this should be in the discussion not the results.

Lines 218-219, unclear statement about detecting center of peaks. Please clarify

Lines 224-225, for a concluding statement, need to explain what favorable operating characteristics means in context.

Line 263, presents is not a great term to use in this context

Line 287, expense of some PR characteristics is unclear and can be better explained with an example.

Line 293, in three CUT&Tag histone modifications, should include the word datasets at the end.

**Authors’ response**

Reviewer 1:

1.Did the authors try to use GoPeaks to call peaks from either ChIP-seq or CUT&RUN datasets? I think it is worthwhile to explore whether it can be applied to these other related techniques. Related, did the authors try to use GoPeaks to call peaks from non- histone modification CUT&Tag (or related) datasets. There is plenty of available CUT&Tag (and other) data for factors such as RNAPII and others. It would be beneficial to the community to report whether GoPeaks can be used for these other datasets.

We agree with the reviewer that it is helpful to understand the extent to which GoPeaks can call peaks from other epigenetic profiling modalities as well as non-histone modification datasets. In response to this comment, we evaluated the ability of GoPeaks to detect peaks from RUNX1 ChIP-seq, Sox2 CUT&RUN, and ATAC-seq data. We found that GoPeaks detected peaks from all three of these epigenetic profiling modalities and, notably, identified regions with a high enrichment of RUNX1 and Sox2 transcription factor motifs from the RUNX1 ChIP-seq and Sox2 CUT&RUN data, respectively (see Figure 8).

2.The authors do a good job with their introduction, although I find many citations missing. As a quick example, in line 73, the authors only cite low input CUT&Tag, when they also mention CUT&RUN, not citing either original (Skene et al 2017) or low input (Skene et al 2018, Hainer et al 2019) CUT&RUN.

We apologize for the omission and agree that it is important to credit authors for their published work. We have updated the introduction with the pertinent citations, including Skene et al 2017, Skene et al 2018, and Hainer et al 2019 (see line 77).

3.It is unclear whether negative control experiments (IgG) are taken into account in GoPeaks analysis.

We apologize for the confusion. The negative control experiments (e.g., IgG, input) are used to scale the sample reads per bin. We provide a more detailed explanation in the methods, including the custom scaling function (see line 404).

4.The authors describe GoPeaks in the first paragraph of the results section. Since the authors then go on to compare GoPeaks performance to MACS2 and SEACR. It would be beneficial to the reader to quickly describe the peak identification process for MACS2 and SEACR for direct comparison.

We agree that it is beneficial to contrast the MACS2 and SEACR methods after describing the GoPeaks peak identification process. Briefly, MACS2 uses a Poisson distribution to estimate peak profiles in the experiment in order to distinguish them from background signal. SEACR bins the genome by regions with contiguous, non-zero signal blocks and sets a peak calling cut-off based on the background signal (see line 129).

5.The authors do a nice job comparing CUT&Tag data to ChIPseq data, such as when described on line 162. However, were the antibodies used the same? This might be important in the experimental data gathered and therefore when comparing peaks from these experiments.

We agree with the reviewer that the antibodies used to generate the CUT&Tag and ChIP-seq data is an important consideration when comparing the peak sets. The antibodies used for the H3K4me1 and H3K27me3 CUT&Tag and ChIP-seq experiments were the same (Abcam ab8895 and Cell Signaling Technology 9733, respectively), but the antibodies for H3K4me3 and H3K27ac were not (Abcam ab8580 and Active Motif 39159 for H3K4me3 ChIP-seq and CUT&Tag, respectively; Millipore 07-360 and Abcam ab4729 for H3K27ac ChIP-seq and CUT&Tag, respectively). We’ve included this consideration in the discussion (see line 383).

6.The authors demonstrate robust results for H3K4me1, H3K4me3, and H3K27ac CUT&Tag data; however these are all euchromatic marks. Does GoPeaks work equally well (or superior to MACS and SEACR) for heterochromatic marks (such as H3K27me3 and H3K9me3)?

We agree with the reviewer that it is important to characterize the extent to which GoPeaks detect heterochromatic as well as euchromatic marks. In response to this comment, we evaluated the ability of GoPeaks to detect H3K27me3 heterochromatic CUT&Tag peaks. We found that GoPeaks and MACS2 have favorable sensitivity and specificity in the identification of H3K27me3 marks. However, MACS2 was not able to retain the broad domain marks characteristic of H3K27me3 and were fragmented the enriched regions of H3K27me3 signal into smaller peaks (see Figure 6 and Supplemental Figure 3).

7.Although certainly up to the authors decision, a web-based served for GoPeaks would broaden appeal and usage by experimentalists, such as what was put together for SEACR.

We agree with the reviewer that it is critical to make these tools accessible and easy- to-use for other scientists. At this point, we are not planning to setup a web server for GoPeaks, but we have made every effort to make our tool intuitive and have posted GoPeaks on Github for anyone to download and use. We are also in the process of uploading the GoPeaks tool as a conda package. 8.There are a few minor typos, such as on line 136 "H3K3me3"

We apologize for the error; this has been corrected (see line 154).

Reviewer 2:

1.In our experience, Cut&Run and Cut&Tag frequently result in a set of background peaks that appear in IgG controls that should be subtracted from final peak sets (like blacklist sites). This manuscript lacks any discussion or information about such controls (with the exception of Figure 3 tracks and legend, but insufficient - was the CBX3 a called peak in IgG?).

We agree with the reviewer that it is important to remove background signal using the IgG or input negative control experiments. We apologize for not making this clearer in the original submission. We remove the background signal by scaling the sample reads per bin proportionally to the reads in the negative control experiments (e.g., IgG, input) in the same bin. Specifically in Figure 3d, GoPeaks did not detect a peak in the CUT&Tag IgG signal at CBX3. We provide a more detailed explanation in the methods, including the custom scaling function (see line 129).

2.It would be useful to provide a brief description of GoPeaks application with other types of data including Cut&Run and ATAC-seq data.

We agree with the reviewer that it is helpful to understand the extent to which GoPeaks detects peaks from other types of epigenetic profiling data. In response to this comment, we evaluated the ability of GoPeaks to detect peaks from RUNX1 ChIP-seq, Sox2 CUT&RUN, and ATAC-seq data. We found that GoPeaks detected peaks from all three of these epigenetic profiling modalities and identified regions with a high enrichment of RUNX1 and Sox2 transcription factor motifs from the ChIP-seq and CUT&RUN data, respectively (see Figure 8).

3.Consistently authors refer to high-confidence peaks as being identified in two biological replicates. First, it isn't clear that these peaks are derived from the intersection or union of the replicates, and second a measure of confidence is generally provided by using the Irreproducibility Discovery Rate (IDR) framework. Authors should clarify the use of replicates to derive 'high-confidence' peak sets and should really consider providing a more robust measure of what is termed high-confidence (i.e. IDR analysis).

We apologize for the confusion and agree with the reviewer that it is important to be clear how replicates were handled. We call high-confidence peaks from our replicates by taking the union of statistically significant peaks from all replicates and retaining the peaks present in at least two biological replicates (see line 146). We agree that the IDR framework is a useful methodology to assess the concordance of peak calls between replicates. However, the IDR framework requires peak p-values to be returned in order to created ranked peak lists. SEACR does not report peak p-values and, therefore, we decided to use our high-confidence method in order to be consistent across all the methods.

4.Similarly, authors also refer to peaks as high-quality peaks (e.g. line 247). This is not clear nor substantiated.

We apologize for the confusion. We have removed this claim from the manuscript (see line 296).

5.The sets of unique peaks identified in Figure 4c should be plotted via signal heatmaps and compared IgG to validate their signal and background.

We appreciate the reviewer’s suggestion to use signal heatmaps to validate the signal of unique peaks as compared to IgG. In response to this comment, we have generated signal heatmaps for all of the histone modification CUT&Tag data (see Supplementary Figures 1-4) as well as for the RUNX1 ChIP-seq and Sox2 CUT&RUN data (see Figure 8). For the sets of unique peaks identified in Figure 4c, we observed that the unique peaks do look enriched as compared to the IgG control (see Supplementary Figure 1c).

6.Line 404, how much antibody was used?

We apologize for the omission. 1 μl of primary antibody was used (see line 498).

7.Line 406, wash buffer references a company but lacks information.

We apologize for the confusion. We make the wash buffer and have included the reagents used to make the buffer in the methods (see line 493).

8.Line 417, 14-27 cycles seems really high.

We agree with the reviewer that 14-27 cycles is higher than usual to prepare CUT&Tag libraries. We used qPCR to determine the optimal number of cycles to run when we generated these CUT&Tag libraries.

9.Figure 4e legend should remove or replace the term regulatory element and put a percentage sign within the barplot.

We apologize for the confusion. We have replaced the term regulatory element throughout the paper with genomic feature (see Figure 4e). We agree that it is important for the readers to understand the percentage of peaks annotated with each genomic feature. Since there are 11 categories of genomic features, a percentage sign on each bar crowds the figures and is particularly difficult to visualize with the thinner bar plots. Therefore, we have included Supplemental Tables with this information so readers can access it (see Supplemental Tables 1 and 2).

10.Line 200, when comparing H3K4me1 peaks to ENCODE data clarify if broad or narrowpeaks were used.

We apologize for the confusion. We have clarified that MACS2 narrow peaks was used throughout the manuscript, except for the H3K27me3 analysis. We used MACS2 broad instead of MACS2 narrow peaks as H3K27me3 peaks can span entire gene bodies (see line 140).

11.Figure 1 represents H3K4me1 enrichment at intergenic regions, however, as discussed in the manuscript, this mark is present in both promoter and distal regions.

Similarly, the Figure 1 legend lacks some information that describes elements of figure (i.e. black box).

We thank the reviewer for pointing out these errors. We have changed Figure 1 so H3K4me1 marks are present in promoter and distal regions and clearly labeled the elements in the figure (see Figure 1).

12.Figure 2 legend mentions subfigure, would be better to use the term box or panel.

We have updated the Figure 2 legend to use the term panel instead of subfigure.

13.Figure 3d gene names should be italicized.

We have italicized the gene name in Figure 3d and ensured that gene names are italicized throughout the manuscript as well.

14.H3K27Ac should be written as H3K27ac, and H3K4Me1, H3K4Me3 (i.e. line 192) should be consistently written as H3K4me1 and H3K4me3.

We thank the reviewer for pointing out these errors. We have updated the manuscript and the figures to consistently use H3K27ac, H3K4me1, and H3K4me3 (see line 208).

15.Introduction paragraph 1 should consider using a single unified description of posttranslational histone modifications, as 3 different description/terms are used:

chemical modifications, post-translation modifications and histone modifications.

We agree with the reviewer that we it is important to make the introduction as clear as possible for readers. We have updated paragraph 1 and replaced removed of chemical or post-translational modifications (see line 56).

16.Line 59 should be nucleosomes not histones.

We thank the reviewer for pointing out this error. We have changed the term to nucleosomes (see line 62).

17.Line 61 states 'binding of these factors' but it isn't clear what is being bound. Please rewrite sentence.

In response to this comment, we have re-written the sentence to clarify that we are referring to transcription factor binding (see line 63).

18.Lines 68 and 296 could also include analysis of other non-histone proteins such as TFs and co-regulators.

We agree with the reviewer. We have clarified that ChIP-seq enables genome-wide profiling of histone modifications and transcription factors (see line 69).

Line 69 implies that ChIP-seq always has high background. However, this is not true.

We agree with the reviewer that ChIP-seq isn’t always associated with high background. We have clarified that while this does not always occur, ChIP-seq is prone to high background particularly compared to methods like CUT&Tag and CUT&RUN (see line 72).

19.Line 139 poorly written statement. Also, would be good to provide brief description of the parameters for comparison (i.e., were they the same thresholds?).

We apologize for the confusion. We have clarified the statement in question (see line 156). Moreover, in response to this comment, we have included a description of the thresholds used for each method (see line 140). For GoPeaks and MACS2, we use a standard FDR threshold of 0.05. For the SEACR methods, we used the SEACR empirically derived FDR threshold

20.Line 142-143, sentence confusing. I would state splitting up enriched regions over splitting up peaks into smaller peaks.

In response to this comment, we have changed the statement to splitting up enriched regions (see line 159).

21.Line 145 to be consistent and clear 10^3 be should be written as 1,000 bp

We appreciate the reviewer for pointing out this error. We have changed 10^3 to 1,000 bp (see line 162).

22.Line 151 should read promoters that were approximately.

We thank the reviewer for pointing out this error. We have changed the statement appropriately (see line 168).

23.Lines 152-153 and other instances (i.e., Lines 190-191, 216-218, etc.) authors discuss and reference biological function of individual genes. However, this is out of context and shouldn't be included in the results. I understand the point is to not miss important peaks, but this should be in the discussion not the results.

We agree with the reviewer that discussing the biological function of individual genes is inappropriate to discuss in the results section. We have removed these statements in the manuscript (see line 169).

24.Lines 218-219, unclear statement about detecting center of peaks. Please clarify

We apologize for the confusion. We have clarified that the center of the peak is the region with the greatest density of histone modification signal (see line 231).

25.Lines 224-225, for a concluding statement, need to explain what favorable operating characteristics means in context.

We apologize for the confusion. We have re-worded this concluding sentence in terms of sensitivity and specificity instead of favorable operating characteristics (see line 207).

26.Line 263, presents is not a great term to use in this context.

We agree with the reviewer and have replaced this term with exhibits (see line 337).

27.Line 287, expense of some PR characteristics is unclear and can be better explained with an example.

We apologize for the confusion. We have removed mentions of PR characteristics and instead presented the findings in terms of sensitivity and specificity (see line 365).

28.Line 293, in three CUT&Tag histone modifications, should include the word datasets at the end.

We thank the reviewer for pointing out this error. We have updated the sentence with the word datasets (see line 370).

In summary, in response to reviewer comments, we evaluated the ability of GoPeaks to detect H3K27me3 heterochromatic CUT&Tag peaks. We found that GoPeaks has favorable sensitivity and specificity in the identification of H3K27me3 marks, while retaining the broad, gene body wide domains. In addition, we measured the ability of GoPeaks to identify peaks from RUNX1 ChIP-seq, Sox2 CUT&RUN, and ATAC-seq data. We found that GoPeaks detected peaks from all three of these epigenetic profiling modalities and, notably, identified regions with a high enrichment of RUNX1 and Sox2 transcription factor motifs from the RUNX1 ChIP-seq and Sox2 CUT&RUN data. Thank you for your thoughtful consideration of our work

**2^nd^ round**

**Reviewer 1**

The authors have done a very thorough job addressing my and the other reviewer comments, and present a strong manuscript that will be beneficial to the community.

**Reviewer 2**

Authors adequately addressed my concerns
