# Supplementary material for: GoPeaks: histone modification peak calling for CUT&Tag
Source: Genome Biol. 2022 Jul 4;23:144. doi: 10.1186/s13059-022-02707-w (PMC9252088; doi:10.1186/s13059-022-02707-w)
Supplement: Supplementary file 2 — Additional file 2: Supplementary Tables S1-2. [file 13059_2022_2707_MOESM2_ESM.docx]

**Fig. S1: GoPeaks demonstrates comparable sensitivity and specificity in identifying H3K4me3 ChIP-seq standard peaks from CUT&Tag data. a, b.** Area under the curve (AUC) of a. ROC and b. PRC from Fig. 4 for each peak calling method. Each bar is labeled by the value it represents. Colors indicate the peak calling method. **c.** Heatmap of global signal from unique H3K4me3 CUT&Tag peaks identified by each method in Fig. 4.

**Fig. S2: GoPeaks demonstrates comparable sensitivity and specificity in identifying H3K4me1 ChIP-seq standard peaks from CUT&Tag data. a, b.** AUC of a. ROC and b. PRC from Fig. 5 for each peak calling method. Each bar is labeled by the value it represents. Colors indicate the peak calling method. **c.** Heatmap of global signal from unique H3K4me1 CUT&Tag peaks identified by each method in Fig. 5. **d.** Comparison of unique peaks that are identified by each peak calling algorithm and are also present in the ChIP-seq standard. Each bar is labeled by the number of peaks it represents. Colors indicate the peak type.

**Fig. S3: GoPeaks demonstrates improved sensitivity and specificity in identifying H3K27me3 ChIP-seq standard peaks from CUT&Tag data. a, b.** AUC of a. ROC and b. PRC from Fig. 6 for each peak calling method. Each bar is labeled by the value it represents. Colors indicate the peak calling method. **c.** Heatmap of global signal from unique H3K27me3 CUT&Tag peaks identified by each method in Fig. 6. **d.** Distribution of read counts by peak width. Each dot represents the read count and peak width of a single detected peak. Colors indicate the peak calling method. **e.** Comparison of unique peaks identified by each peak calling algorithm and how many are also present in the ChIP-seq standard. Each bar is labeled by the number of peaks it represents. Colors indicate the peak type.

**Fig. S4: GoPeaks demonstrates improved sensitivity and specificity in identifying H3K27ac ChIP-seq standard peaks from CUT&Tag data. a, b.** AUC of a. ROC and b. PRC from Fig. 7 for each peak calling method. Each bar is labeled by the value it represents. Colors indicate the peak calling method. **c.** Heatmap of global signal from unique H3K27ac CUT&Tag peaks identified by each method in Fig. 7. **d.** Comparison of unique peaks identified by each peak calling algorithm and how many are also present in the ChIP-seq standard. Each bar is labeled by the number of peaks it represents. Colors indicate the peak type. **e.** Distribution of read counts by peak width. Each dot represents the read count and peak width of a single detected peak. Colors indicate the peak calling method.
